# Editorial: Research progress on immune microenvironment and molecular mechanism of glioma

**DOI:** 10.3389/fonc.2023.1288309

**Published:** 2023-10-12

**Authors:** Hany E. Marei, Liang Wang, Carlo Cenciarelli, Wei Zhang, Wei Hua

**Affiliations:** ^1^Department of Cytology and Histology, Faculty of Veterinary Medicine, Mansoura University, Mansoura, Egypt; ^2^Tangdu Hospital, Fourth Military Medical University, Xi’an, China; ^3^Institute of Translational Pharmacology, Italian National Research Council (IFT)-CNR, Rome, Italy; ^4^Beijing Neurosurgical Institute, Beijing Tiantan Hospital, Capital Medical University, Beijing, China; ^5^Huashan Hospital, Fudan University, Shanghai, China

**Keywords:** heterogeneity, glioma, immune microenvironment, immunotherapy, diagnosis, molecular mechanism

The most frequent primary intracranial tumour, glioma causes severe morbidity and mortality. The survival rate is still dismal despite substantial research improvements in molecular processes, diagnostics, and therapy strategies. The occurrence and progression of gliomas as well as the results of anti-tumor immunotherapy are all directly tied to the very varied immune microenvironment of gliomas. Numerous chemokines, cytokines, and growth factors secreted by glioma cells encourage the microenvironment infiltration of different immune cells like macrophages, myeloid-derived suppressor cells, CD4+ T cells, and Treg. These cytokines and chemokines, as well as their interactions with the extracellular matrix, alter the functional phenotypes of infiltrating immune cells, steering the immune system to speed up the evolution of gliomas and make anti-immunotherapy more effective.

The diversity of molecular subtypes is frequently related to patient treatment and prognosis, according to earlier studies on the molecular mechanism of glioma. Higher levels of EGFR amplification, TERT promoter mutations, and PTEN loss are typically found in IDH-Wild Type (WT) glioblastomas. About half of the IDH-WT glioblastomas, with a better prognosis and responsiveness to treatment, have MGMT promoter methylation. Low-grade gliomas in children and adults have various genetic characteristics; low-grade gliomas in children often have FGFR1 and BRAF mutations, whereas low-grade gliomas in adults usually have IDH1/2 and ATRX mutations, occasionally with 1p/19q co-deletions oligodendroglioma. These molecular characteristics can be used to better categorise gliomas, determine an individual patient’s course of treatment, and improve prognosis, although standard treatments are currently only successful for those patients with glioblastoma (GBM) who have MGMT promoter methylation. Therefore, research into the molecular mechanisms of gliomagenesis and the modulate of the glioma microenvironment will not only aid in a better understanding of these mechanisms but also aid in the development of precise targeted therapies and immunotherapy techniques, as well as improve the sensitivity of glioma chemotherapy. In order to develop strategies that could target the expression of glioma molecular markers and regulate the remodelling of the immune microenvironment, as well as provide useful references for enhancing immunotherapy and targeted therapy, this Research Topic aims to shed light on the mechanism of the heterogeneity of the glioma immune microenvironment and the mechanism that affects glioma progression and treatment resistance, including low-grade gliomas in paediatrics and adults.

This Research Topic includes 8 manuscripts that highlight current knowledge and future directions for the role of immune microenvironment and molecular mechanism of glioma.

In high-grade gliomas, reduced expression of TSPAN7 was linked to a poor prognosis in glioma patients, according to Chen et al. TSPAN7 expression in glioma clinical samples and glioma cell lines was also confirmed by qPCR, Western Blotting, and immunofluorescence. Additionally, functional enrichment analysis revealed that the TSPAN7 lower expression subgroup had activated cell proliferation, EMT, angiogenesis, DNA repair, and MAPK signalling pathways. To investigate TSPAN7’s potential anti-tumor properties in glioma, lentiviral plasmids were employed to overexpress it in the U87 and LN229 glioma cell lines. Additionally, the authors discovered that TSPAN7 was strongly adversely linked with the immunological infiltration of tumor-related macrophages, particularly M2-type macrophages, by examining the association between TSPAN7 expression and the immune cell infiltration across several datasets. Further examination of immunological checkpoints revealed a negative correlation between TSPAN7 expression and that of PD-1, PD-L1, and CTLA-4. The authors showed that TSPAN7 expression may have a synergistic effect with PD-L1 on the responsiveness to anti-PD-1 immunotherapy in a GBM cohort. They hypothesized that TSPAN7 can function as a biomarker for prognosis and as a potential immunotherapy target in glioma patients based on the aforementioned findings.

According to Tang et al., human glioma models are essential for both enhancing our understanding of glioblastoma biology and facilitating the creation of therapeutic approaches. It has been difficult to create lower-grade glioma (LGG) models, which has led to a dearth of studies and no advancement in conventional treatment during the past ten years. However, LGG models must follow precise guidelines that accurately represent tumour genetic aberrations and the microenvironment in order to predict and validate the efficacies of innovative treatments. This emphasises the necessity to review current LGG models and investigate prospective models that might close the gap between preclinical understanding and clinical translation. The first section of this review presents a set of standards intended to solve the current issues impeding model development. The authors then assess the advantages and disadvantages of the current preclinical LGG models in relation to these norms. In order to maximize the investigation of disease causes and the development of new treatments, the study concludes by discussing potential future approaches for merging existing models.

Four molecular subgroups of glioma were discovered, according to Zhang et al., with the C1 group having the worst prognosis. Results from Principal Component Analysis (PCA) and heatmaps showed that transmembrane protein genes associated with prognosis displayed differential expression in all four groups. Additionally, there was a large amount of variation in the four groups’ microenvironments. The 6-gene-based signature may forecast a patient’s overall survival (OS) at 1, 2, and 3 years. The signature outperformed conventional clinical factors and could be utilised as a standalone predictor of glioma OS. The high-risk group exhibited a higher influx of immune cells, indicating immune escape. The investigators gene signature revealed that a large number of genes were related to the composition of immune cells, suggesting that transmembrane protein-related genes may affect the growth and prognosis of glioma through influencing the immunological milieu. The random forest algorithm determined that TMEM158 was the most significant gene signature. Multiple malignant cells expressed TMEM158, as evidenced by the single-cell datasets.

By controlling tumour progression in numerous ways, the expression of transmembrane protein-related genes will be tightly correlated with the immunological state and prognosis of glioma patients. Future research on the molecular mechanism and targeted treatment of glioma is made possible by the interplay between transmembrane protein-related genes and immunity during the development of glioma.

According to Zhang et al., astrocytes make up around 30% of the cells in gliomas and are crucial for the development and survival of synapses. A new form of astrocyte with activation of the JAK/STAT pathway have been recently discovered. What these tumor-associated reactive astrocytes (TARAs) in gliomas mean is still a mystery. By examining five different datasets, the authors thoroughly evaluated TARAs in gliomas, both in individual cells and at the level of the entire tumour. First, they calculated the degree of TARA infiltration in gliomas using two single-cell RNA sequencing datasets containing 35,563 cells from 23 individuals.

In order to assess the genomic, transcriptomic, and clinical characteristics of TARA infiltration, they also gathered clinical information, genomic data, and transcriptomic data from 1,379 diffuse astrocytoma and glioblastoma samples from the Chinese Glioma Genome Atlas (CGGA) and The Cancer Genome Atlas (TCGA) datasets. Third, they examined the predictive usefulness of TARAs for immune checkpoint inhibition by downloading expression profiles of recurrent glioblastoma samples from patients receiving PD-1 inhibitors. TARAs were prevalent in the glioma microenvironment, according to single-cell RNA sequencing data (15.7% in the CGGA dataset and 9.1% in the Gene Expression Omnibus GSE141383 dataset, respectively). The level of TARA infiltration was strongly correlated with the main clinical and molecular characteristics of astrocytic gliomas, according to bulk tumour sequencing data. Patients who had higher TARA infiltration were more likely to have chromosomal 9p21.3, 10q23.3, and 13q14.2 deletions, as well as MUC16, FLG, and PICK3A mutations and 7p11.2 amplification. The high level of astrocyte infiltration, as revealed by gene ontology analysis, was associated with immune and oncogenic pathways, including the inflammatory response, positive regulation of the JAK-STAT cascade, positive regulation of NIK/NF-kappa B signalling, and the biosynthesis of tumour necrosis factor. Patients’ prognoses were worse in those with more TARA infiltration. The degree of reactive astrocyte infiltration, meantime, demonstrated a prognostic value for individuals with recurrent glioblastoma receiving anti-PD-1 immune treatment. TARA infiltration may accelerate the growth of glioma tumours and is a useful diagnostic, prognostic, and predictive marker for gliomas. A novel therapeutic approach for glioma might involve preventing TARA invasion.

The most frequently altered gene in glioblastoma (GBM) is the epidermal growth factor receptor (EGFR), which is crucial for the growth of the tumour and the anti-tumor immune response, according to (Li et al.). However, current molecular targeted therapeutics against the EGFR signalling system and its essential downstream molecules have not shown promising clinical results in GBM. On the contrary, tumour immunotherapies, particularly immune checkpoint inhibitors, have demonstrated long-lasting anticancer effects in a variety of malignancies. The poor clinical efficacy in patients with EGFR mutations, however, suggests that tumour immune response may play a role in EGFR signalling. According to recent research, EGFR mutations not only encourage the growth of GBM cells but also have an impact on the immune system in the tumour microenvironment (TME), attracting immune suppressive cells such M2-like TAMs, MDSCs, and Tregs, and preventing T and NK cell activation. The expression of immunosuppressive molecules or cytokines (such PD-L1, CD73, and TGF-β is also upregulated by EGFR changes. In order to build a theoretical foundation for combining targeted EGFR inhibitors with immunotherapy for GBM, this paper examines how EGFR mutations contribute to the development of an immunosuppressive TME.

Gliomas are distinguished by their immunosuppressive characteristics and therefore poor patient prognosis, according to (Tao et al.). According to recent research, the tumour microenvironment plays a critical role in the development of gliomas. This role is largely attributed to tumor-associated macrophages, such as brain-resident microglia and bone marrow-derived macrophages, which foster the growth and invasion of tumour cells. But it’s still difficult to tell these two cell subgroups apart. In order to better understand the complicated interactions between microglia and glioma cells, this review first analyses the heterogeneity between these two cell types. The authors follow with an overview with current immunotherapy initiatives that focus on microglia. However, as independent research on microglia is still in its early stages and currently has numerous inadequacies, the authors highlight their related concerns and hope that additional study will be conducted in the future to solve these problems.

Angiopoietins (ANGs), stromal-derived factor-1 (SDF-1), platelet-derived growth factors (PDGFs), fibroblast growth factors (FGFs), transforming growth factor-beta (TGF-), and vascular endothelial growth factors (VEGFs) are among the major members of the class of secreted cytokines known as angiogenic growth factors (AGFs) that are related to angiogenesis. A growing body of research suggests that AGFs have a role in the growth of tumours through mechanisms other than their ability to promote angiogenesis. In the glioma microenvironment, which is characterised by significant angiogenesis and severe immunosuppression, AGFs were found to be increased. By interacting with immune cells, AGFs produced by tumour and stromal cells can have an immunomodulatory effect in the glioma microenvironment. With a focus on glioma, this review summarised the interactions between AGFs, immune cells, and cancer cells. It also offered fresh viewpoints for comprehending the glioma immune microenvironment and in-depth investigations for anti-glioma therapy (Ge et al.).

According to Yang et al., saffron is an active ingredient in traditional Tibetan medicine (TTM), which may have anticancer properties. Here, the investigators looked at safranal’s therapeutic impact and workings on GBM.

Safranal suppressed GBM cell proliferation and invasion *in vitro*, as demonstrated by CCK-8, GBM-brain organoid coculture tests, and 3D tumour spheroid invasion assays. Safranal was predicted and found to be able to increase GBM cell death, G2/M phase arrest, and inhibit the PI3K/AKT/mTOR axis using network pharmacology, RNA-seq, molecular docking analysis, western blotting, and cell cycle experiments. Safranal can both reduce GBM cell growth alone and when combined with temozolomide (TMZ), according to *in vivo* tests.

This study showed that safranal enhances GBM cell apoptosis and G2/M phase arrest, inhibits the PI3K/AKT/mTOR axis, and can enhance the efficacy of TMZ. It also showed that safranal suppresses GBM cell growth *in vivo* and *in vitro*.

## Author contributions

HM: Writing – original draft. LW: Writing – original draft, Writing – review & editing. CC: Writing – original draft, Writing – review & editing. WZ: Writing – original draft, Writing – review & editing. WH: Writing – original draft, Writing – review & editing.

